# Molecular characterization of two new alternaviruses identified in members of the fungal family Nectriaceae

**DOI:** 10.1007/s00203-023-03477-0

**Published:** 2023-03-22

**Authors:** Tom P. Pielhop, Carolin Popp, Sebastian Fricke, Dennis Knierim, Paolo Margaria, Edgar Maiß

**Affiliations:** 1 Institute of Horticultural Production Systems, Department Phytomedicine, Leibniz University Hannover, Herrenhäuser Str. 2, 30419 Hannover, Germany; 2 Leibniz Institute DSMZ, German Collection of Microorganisms and Cell Cultures, Inhoffenstraße 7 B, 38124 Braunschweig, Germany

**Keywords:** Mycovirus, Alternavirus, dsRNA, *Dactylonectria torresensis*, *Ilyonectria robusta*, Apple replant disease, RNA capping

## Abstract

Since the first report in 2009, at least ten additional viruses have been identified and assigned to the proposed virus family *Alternaviridae*. Here we report two new mycoviruses tentatively assigned to this family, both identified as members of the fungal family Nectriaceae, which were isolated from surface-disinfected apple roots (*Malus x domestica*, Borkh.) affected by apple replant disease (ARD). ARD is a highly complex, worldwide-occurring disease resulting from plant reactions to a disturbed (micro)-biome and leads to high economic losses every year. The first alternavirus characterized in this study was identified in a *Dactylonectria torresensis* isolate. The virus was tentatively named dactylonectria torresensis alternavirus 1 (DtAV1) as the first member of the proposed new species *Alternavirus dactylonectriae*. The second virus was identified in an isolate of *Ilyonectria robusta* and was tentatively named ilyonectria robusta alternavirus 1 (IrAV1) as the first member of the proposed new species *Alternavirus ilyonectriae*. Full genomic sequences of the viruses were determined and are presented. Further, we found hints for putative components of a methyl transferase machinery using in silico approaches. This putative protein domain is encoded by segment 2. However, this result only establishes the basis for subsequent studies in which the function must be confirmed experimentally in vitro. Thus, this is the first study where a function is predicted to all three genomic segments within the group of the alternaviruses. These findings provide further insights into the virome of ARD-associated fungi and are therefore another brick in the wall of understanding the complexity of the disease.

**Supplementary Information:**

The online version contains supplementary material available at 10.1007/s00203-023-03477-0.

## Introduction

Mycoviruses are the subject of research in the virology field since their first description in 1962 (Hollings 1962). In recent years, especially since HTS techniques have become affordable for many research institutions worldwide, an increasing number of new viruses are continuously being assigned to the individual mycoviral taxa. Meanwhile, viral infections have been detected in all major fungal taxa (Ghabrial et al. 2015; Pearson et al. 2009). 

Most mycoviruses possess a dsRNA or + ssRNA genome. Just a few -ssRNA or DNA viruses are known to infect fungi (Khalifa and MacDiarmid 2021; Kotta-Loizou 2021; Yu et al. 2010). For the vast majority of mycoviruses, no direct impact on their host could be shown so far. However, when observing viral infections of (phyto)-pathogenic fungi, some viruses were found to have either a hypo- or hypervirulent impact on their host (Kotta-Loizou 2021; Deng et al. 2003; Nuss 2005; Olivé and Campo 2021; Özkan and Coutts 2015; Polashock and Hillman 1994).

Here, we report the identification of two new mycoviruses, assigned to the proposed family *Alternaviridae*. Both were identified in dsRNA extracts from fungi assigned to the family Nectriaceae. The first virus belongs to the virome of a *Dactylonectria torresensis* isolate and was tentatively named dactylonectria torresensis alternavirus 1 (DtAV1), as a member of the proposed new virus species *Alternavirus dactylonectriae*. Secondly, we report a virus identified in an *Ilyonectria robusta* isolate, which was tentatively named ilyonectria robusta alternavirus 1 (IrAV1) as a member of the proposed new species *Alternavirus ilyonectriae.*

Up to now, eleven viruses have been assigned to the proposed family *Alternaviridae*. The eponymous alternaria alternata virus 1 (AaV-1) was identified in 2009 and the previously found aspergillus mycovirus 341 (AMV) was assigned to the family of which nine more viruses were discovered all around the world in the following years (Aoki et al. 2009; Gilbert et al. 2019; Hammond et al. 2008; He et al. 2018; Kozlakidis et al. 2013; Osaki et al. 2016; Wen et al. 2021; Zhang et al. 2019, 2022; Lutz et al. 2022). One of the most prominent features of this family is the ADD sequence triplet (motif VI) in the RdRp amino acid (aa) sequence. This motif is highly conserved among all RNA viruses and usually consists of a GDD (or less frequent SDD) triplet (Koonin 1991; Kamer and Argos 1984).

Both viruses characterized in this work were identified in members of the fungal family Nectriaceae. The fungi were in turn isolated and identified as part of the endophytic microbiome of apple plants (*Malus x domestica*, Borkh.) affected by apple replant disease (ARD). ARD is a worldwide problem, occurring when apple plants are replanted repeatedly in the same site and are thought to be caused by plant reactions due to a disturbed (micro)-biome (Winkelmann et al. 2019). The disease can lead to losses of up to 50% of the profitability of apple orchards by reduction of yield and a delay of the plants in bearing fruits (Schoor et al. 2009). Up to now, the disease etiology is still unclear. However, fungi of the family Nectriaceae were found repetitively at high abundance in suffering apple plants and seem to influence the disease expression (Manici et al. 2018; Popp et al. 2019).

Since there is still no effective and sustainable control strategy against ARD, all involved biotic factors like nematodes, bacteria, oomycetes, and fungi have to be investigated in detail. As part of the virome of associated endophytic fungi, the mycoviruses described in this work may play a role in the occurrence of the disease. This study is thus the basis for further analyses to evaluate the targeted deployment of mycoviruses in control strategies to counteract the ARD.

## Materials and methods

### Fungal material

Both fungal isolates investigated in this study were obtained among others in a central experiment in 2017 from apple roots, grown in ARD soil (Mahnkopp et al. 2018). The *D. torresensis* isolate O6-1-A and the *I. robusta* isolate O16-2-D were isolated from plants grown in soil from the experimental sites Ellerhoop, (53.714361, 9.770139; Schleswig–Holstein, Germany) and Heidgraben (53.699199, 9.683171; Schleswig–Holstein, Germany). Plant roots were surface disinfected and subsequently plated on water agar with rifampicin (25 µg mL^−1^), penicillin (50 µg mL^−1^), and pimaricin (25 µg mL^−1^). Outgrown fungi were picked and cultured on malt extract agar (MEA, 2%).

Fungi are usually identified by ITS-PCR and BLAST. Since this method is insufficient for the accurate identification of members of the Nectriaceae family, multi-locus analyses were performed (Lawrence et al. 2019; Cabral et al. 2012). O6-1-A was identified as *D. torresensis* by amplification of the translation elongation factor 1-α (TEF) and histone 3 (HIS) genes. TEF was amplified with the primer pair CylEF-1 (Groenewald, J. Z., unpublished) and CylEF-R2 (Crous et al. 2004). For the identification by HIS, the primers CYLH3F and CYLH3R were used (Crous et al. 2004). O16-2-D was identified as *I. robusta* by amplification of three loci. In addition to TEF and HIS, the β-tubulin gene (TUB) was analyzed with the primers T1 and Bt2b (O'Donnell and Cigelnik 1997; Glass and Donaldson 1995). All primers used for fungal identification are summed up in Table 1. PCR-fragments were purified, sent to Microsynth SeqLab (Goettingen, Germany) for Sanger sequencing, and the sequences were analyzed by NCBI BLASTn, subsequently (Popp et al. 2019; Sanger et al. 1977).

**Table 1:** Primers used to identify the fungal isolates by multi-locus analysis

Primer No	Designation	Lit	Target region	Sequence 5′-3′
1	CylEF-1	[Groenewald, J.Z., unpublished]	TEF sense	ATGGGTAAGGAVGAVAAGAC
2	CylEF-R2	[Crous et al. 2004]	TEF antisense	CATGTTCTTGATGAA(A/G)TCACG
3	CYLH3F	[Crous et al. 2004]	HIS sense	AGGTCCACTGGTGGCAAG
4	CYLH3R	[Crous et al. 2004]	HIS antisense	AGCTGGATGTCCTTGGACTG
5	T1	[O'Donnell and Cigelnik 1997]	TUB sense	AACATGCGTGAGATTGTAAGT
6	Bt2b	[Glass and Donaldson 1995]	TUB antisense	ACCCTCAGTGTAGTGACCCTTGGC

### Nucleic acid extraction

To obtain enough material for nucleic acid extraction, fungi were grown in 2% malt extract broth for two weeks before the mycelium was filtered and ground in liquid nitrogen. DsRNA was extracted from 20 g ground mycelium with a modified protocol, based on the method published by Morris and Dodds in 1979 as described in Lesker et al. 2013 except for the use of a different cellulose (Merck; Darmstadt, Germany; product nr. 22,184) (Morris and Dodds 1979; Lesker et al. 2013). 20 U RNase T1 and 40 U DNase I (both: Roche; Basel, Switzerland) were used to digest 20 mL eluate at 37 °C for 30 min each. The dsRNA extracts were purified and concentrated in a 3 mL storage volume. 500 µL eluate were precipitated with ethanol and solved in 20 µL water for subsequent analyses. The purified dsRNA extracts were used for the identification of viral segments (S1–S3) by agarose gel electrophoresis and subsequent Illumina sequencing at the Leibniz Institute DSMZ (Brunswick, Germany).

RT-PCRs for virus detection, end determination by poly (A) tail hybridization, and 5′-RACEs (rapid amplification of cDNA ends) were performed with whole nucleic acid extractions. Those extractions were done with the same ground fungal material as for dsRNA extraction. The methodology for the whole nucleic acid extractions and purification was obtained from a simpler protocol as described in (Menzel et al. 2002).

### High-throughput sequencing and bioinformatics analysis

The isolated dsRNA was used as input for cDNA synthesis with random octamer primers and the Maxima H Minus Reverse Transcriptase (Thermo Fisher Scientific, Waltham, USA), followed by second-strand synthesis and library preparation according to the Nextera XT DNA Library Preparation Kit (Illumina; San Diego, USA). Sequencing of the libraries was performed at the Leibniz-Institute DSMZ on a MiSeq instrument as paired-end reads (2 × 301 bp). Raw reads were trimmed and de novo assembled with the Geneious Prime^®^ software (Biomatters, Auckland, New Zealand) using an in-house established workflow, and putative virus contigs were identified by BLASTn/BLASTp alignment against a custom database of NCBI nuclear-core reference sequences. The contigs were subsequently ordered and trimmed according to reference sequences. The resulting sequence information was used to design primers for validation and determination of full-length genome sequences by RT-PCR and RACE reactions.

### RT-PCR and RACE

To detect the viral segments in fungal subcultures, to confirm the HTS results, and assemble full-length genomes, RT-PCRs with specific primers were developed for each virus. A cDNA synthesis mix was set up with 3 µL whole nucleic acid extract, 1 µL antisense identification primer for the respective viral segment (primer no.: 8, 10, 12, 23, 25, 27, Table 2), 0.5 µL dNTPs (10 mM each; Carl Roth; Karlsruhe, Germany), 2 µL RT buffer, and 20 U RevertAid reverse transcriptase (both: Thermo Fisher Scientific, Waltham, USA) in a total volume of 10 µL. cDNA synthesis was performed at 42 °C for 1 h. Subsequent PCR set-up with 5 µL Phusion Flash High-Fidelity PCR Master Mix (Thermo Fisher Scientific, Waltham, USA), 0.5 µL of each forward and reverse identification primer for the respective viral segment (primer no.: 7–12, 22–27; Table 2) and 1 µL cDNA from the previous step, in a final volume of 10 µL. The PCR program consisted of a denaturation step of 15 s at 98 °C, followed by 34 cycles with a 15 s denaturation at 98 °C, 5 s primer hybridization at the respective annealing temperature (T_A_), and 20 s elongation at 72 °C. A final elongation was performed at 72 °C for 5 min.

**Table 2:** Primers used for detection- and identification-PCRs and end determination by RACE

Primer No	Designation	Sequence 5′ 3′
7	DacRNA1_ident_s	CGTATGAAGAACTGTTGGCTACCCG
8	DacRNA1_ident_as	CGACATCATCAGCACGATTGAGGG
9	DacRNA2_ident_s	CCGTGCCTTAACAAGCCTGGG
10	DacRNA2_ident_as	GCCTTGTCAGCAGATCCATGCC
11	DacRNA3_ident_s	CGCTTTCATGCCATCGGTGAG
12	DacRNA3_ident_as	CGGAAATCATTGACACCACGACC
13	Dac_1_5end	CGCTGCGCACCAACAAATTC
14	Dac_2_5end	GACGGCTACCGAGAGGAAGTTAGC
15	Dac_3_5end	GTACACCTCGTGCGTAGGATCG
16	Dac_1_5_nested	TCCTCCGCTTATTGATATGC
17	Dac_2_5_nested	TTGATATAGGACACCTTGCCAGTCTGAG
18	Dac_3_5_nested	TCGGCCTGATCAGGCATTTTGAAC
19	Dac_1_3_end	GCATGGGACAAGTTGATACCGC
20	Dac_2_3_end	CGTACTTGCAGCCGCCAACGC
21	Dac_3_3_end	GATGCGGCGTACCGTGCATCG
22	IlyRNA1_ident_s	TACGTGTTCAAGCACTTTAGC
23	IlyRNA1_ident_as	CTATCGGTATGGTCGACACGG
24	IlyRNA2_ident_s	GACTCATCTGTCTGGAATTACGGAATGGTG
25	IlyRNA2_ident_as	CACTACTGTCCTATCAGTATCCAACTCAGC
26	IlyRNA3_ident_s	TTGGCGCTATCGTGCTCCGAACGACCCAC
27	IlyRNA3_ident_as	CGAAAGTAATCCCGGTCAAGTACGGTTCTGCC
28	Ily_1_5end	GTCACCGTCGTATACAACGACTG
29	Ily_2_5end	CCAAATGCAACGCGATCTCAACT
30	Ily_3_5end	GGGTTGACGAGCGTCTATGTCGC
31	Ily_1_5_nested	GTGCAGCTGTATCAGAAGTGACA
32	Ily_2_5_nested	CCCAACTACCATCGTACGAGTAG
33	Ily_3_5_nested	ACATTGTGCCTGGTATCACTGC
34	Ily_1_3_end	GAGTGGTCTCTCGAAGCACGCA
35	Ily_2_3_end	TCTGAACGATCATTCGGCTCGCA
36	Ily_3_3_end	ACTCTCCGCGGTTCTGAGACTG
37	Poly-G15	CTCAAACAGTCACGGGGGGGGGGGGGGG
38	Poly-C14	ATCCTGCAGGCGCGCCCCCCCCCCCCCC
39	RACE-BOE1	GACCACGCGTATCGATGTCGACTTTTTTTTTTTTT TTT(AGC)
40	RACE-BOE2	GACCACGCGTATCGATGTCGA

The 5′ ends of the six viral segments were determined with a modified RACE protocol, based on the method described by Frohman et al. (Frohman et al. 1988). The cDNA used for the RACE was synthesized as described for the RT-PCRs apart from using different primers (primer no.: 13–15; 28–30; Table 2). After purifying with SureClean Plus (Bioline; London, UK), 3 µL cDNA was tailed in two reactions with 1 µL of either dGTP or dCTP (100 mM; Thermo Fisher Scientific; Waltham, USA) in a mix with 1 µL terminal deoxynucleotidyl transferase (TdT; 20 U mL^−1^), 4 µL 5 × TdT Reaction Buffer (both: Thermo Fisher Scientific; Waltham, USA) and 11 µL water at 37 °C for 30 min, followed by an inactivation step for 10 min at 70 °C. The subsequent PCR setup was identical to the RT-PCR described above, aside from using the respective nested primers (primer no.: 16–18; 31–33; Table 2) and the corresponding poly (G) or poly (C) primer (primer no.: 37, 38; Table 2).

Determination of 3′-ends was done by detecting the poly (A) tail that all segments have in common. A detectable linker was hybridized to each poly (A) tail by cDNA synthesis with the RACE-BOE1 primer (primer no. 39; Table 2) in a mixture as described above. The following PCR was set up with the corresponding RACE-BOE2 primer (no. 40; Table 2) and the specific 3′ primer (no.: 19–21; 34–36; Table 2) in a mixture as described for the detection PCR.

### Alignments of UTRs and amino acid motifs

5′ ends of the three segments of both DtAV1 and IrAV1 were compared to identify conserved regions. Alignments were performed in MEGA X using the MUSCLE algorithm (Edgar 2004; Kumar et al. 2018). The GeneDoc software (National Resource for Biomedical Supercomputing, Pittsburgh, USA) was used to visualize the RNA alignments. To calculate aa alignments and identify conserved domains within the RdRps and methyltransferases (MTase), the Clustal Omega algorithm was used (Sievers et al. 2011). Conservation indices were calculated and annotated according to the Gonnet PAM250 matrix (Gonnet et al. 1992).

### Phylogenetic analyses

To perform comparative phylogenetic analyses, all available RdRp and CP amino acid sequences of members assigned to the proposed family *Alternaviridae* were compiled. Since the CP was not yet annotated for most of the viruses within this group, comparative BLASTp searches were performed and the respective viral segments were assigned to their function as major CP according to the protein determination of (Wu et al. 2021). Penicillium chrysogenum virus (PcV) was chosen as an outgroup for the phylogenetic trees since it has been shown that chrysoviruses are closely related to alternaviruses (Aoki et al. 2009; Castón et al. 2003). The set of RdRp sequences was composed of 14 different proteins. The set of CP sequences comprised 13 sequences since segment 1 (S1) is the only available sequence of AMV. Respective sequence sets were aligned using the MUSCLE algorithm with default settings (gap opening: −2.9, gap extension: 0) in MEGA X (Edgar 2004; Kumar et al. 2018). Best-performing substitution models were calculated for both alignments. For the RdRp-based tree, the Le_Gascuel_2008 was determined as the best model with gamma-distributed rates and empirical base frequencies (LG + G + F) (Le and Gascuel 2008). The calculations for the CP-tree are based on the empirical model by Whelan and Goldman with gamma-distributed rates and base frequencies (WAG + G + F) (Whelan and Goldman 2001). Finally, Maximum Likelihood trees were calculated using the bootstrap method with 1000 replicates and all sites from the data subsets. Moreover, pairwise sequence identity matrices we calculated comparing the RdRP and CP sequences of all available alternaviruses using the EMBOSS/Needle tool with default settings (Madeira et al. 2019).

### Protein folding modeling and function prediction

To get hints on the function of the protein encoded by dsRNA2, the protein folding was predicted by using the ColabFold code, which is available for free as a GoogleColab worksheet (Mirdita et al. 2022). With this worksheet, protein structures are predicted by using AlphaFold2 and the Alphafold2-multimer (Jumper et al. 2021; Evans et al. 2021). Hints on protein functions were subsequently calculated by using the structure-based function tool DeepFRI (Gligorijević et al. 2021). After the identification of high-scoring functional domains, respective regions of the amino acid sequence were analyzed using the homology modeling service of the SWISS-MODEL server to confirm the domains by comparison with PDB-deposited templates and specify their putative function (Waterhouse et al. 2018).

## Results and discussion

### Discovery of two new alternaviruses

**Fig. 1:**
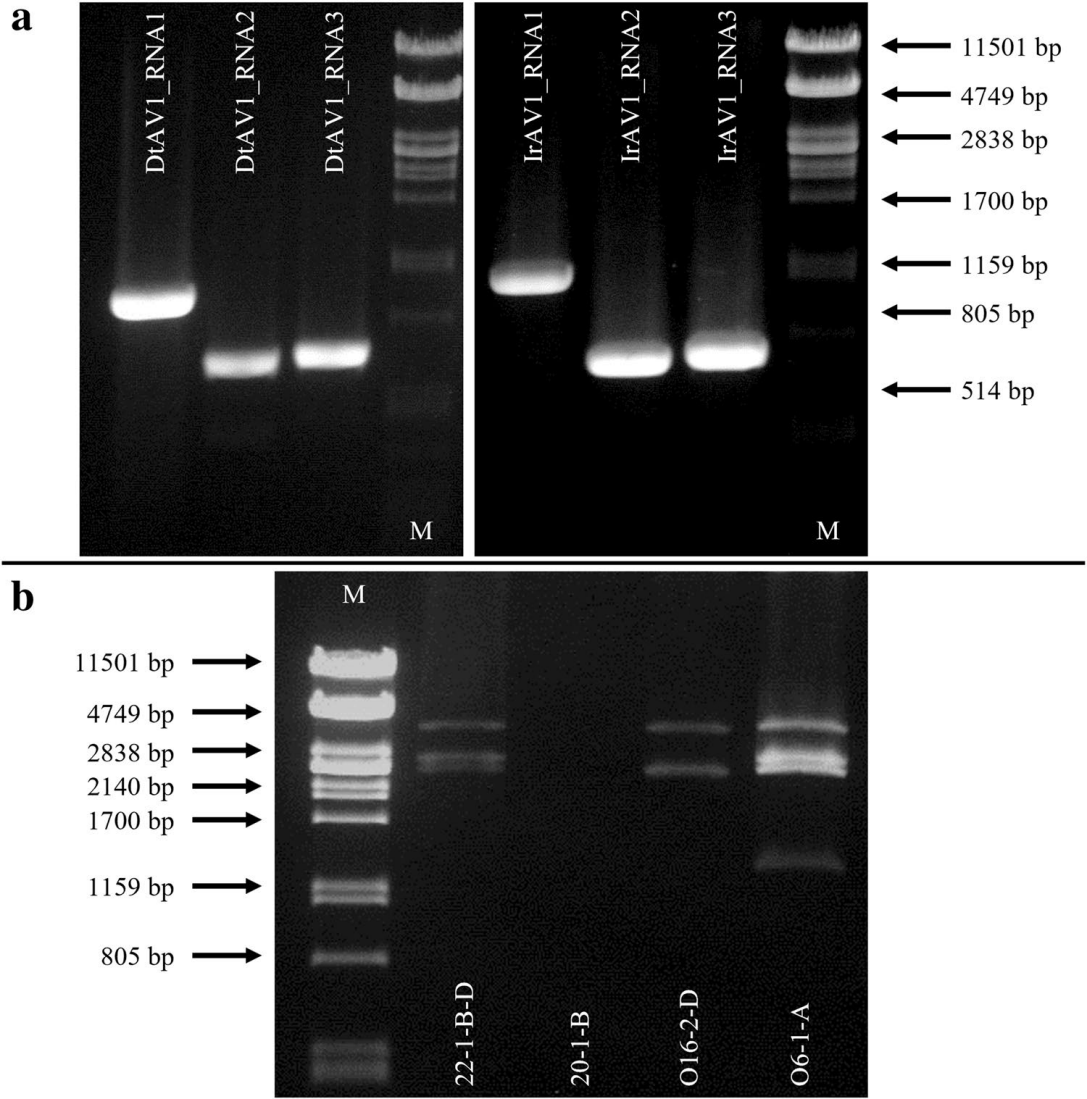
Agarose gel electrophoreses: **a** Detection RT-PCR of DtAV1 (left) and IrAV1 (right). Marker (M): PstI digested Lambda-Phage DNA, Polymerase: PhusionFlash (ThermoScientific™), Primers according to Table 2: DtAV1_RNA1: 7 + 8; DtAV1_RNA2: 9 + 10; DtAV1_RNA3: 11 + 12; IrAV1_RNA1: 22 + 23; IrAV1_RNA2: 24 + 25; IrAV1_RNA3: 26 + 27; Primer concentration: 10 µM. **b** dsRNA-extractions of 22–1–B–D, 20–1–B (*D. torresensis* isolates irrelevant to this study), O16–2–D (*I. robusta*) and O6-1-A (*D. torresensis*), Marker (M): PstI digested Lambda-Phage DNA

Gel electrophoresis of the dsRNA extracts of *Dactylonectria torresensis* O6-1-A and *Ilyonectria robusta* O16-2-D revealed four and two fragments, respectively. For *D. torresensis*, the sizes of the fragments were estimated to be 3600 bp, 2700 bp, 2500 bp, and 1300 bp, and for *I. robusta* about 3600 bp and 2500 bp. Both dsRNA extractions are shown in Fig. 1b, together with two additional *D. torresensis* isolates (22-1-B-D, 20-1-B). Both of these isolates (22–1–B–D and 20–1–B) were not part of this study and are therefore negligible.

Illumina sequencing of the libraries generated 408.650 and 473.416 reads from samples DtO6-1-A and IrO16-2-D, respectively, and the bioinformatic analyses revealed a number of contigs which were assigned to putative alternaviruses (Online Resource Table ESM1). To validate the results, all of the sequences were subsequently confirmed by RT-PCRs in the respective hosts, and 5′- and 3′-ends were determined by RACE to assemble the full-length genomic sequences.

The three virus genome segments assembled from *D. torresensis* have a length of 3578 bp, 2668 bp, and 2467 bp, which correlated to the size of the three larger fragments visible on the gel (Fig. 1b). These segments were assigned to a new member of the proposed family *Alternaviridae*, tentatively named dactylonectria torresensis alternavirus 1 (DtAV1) being the first member of the proposed new species *Alternavirus dactylonectriae.* A fourth fragment which was visualized by electrophoresis and putatively assigned to a 4th RNA of DtAV1 was not identified in the bioinformatics analysis by homology search against the segment 4 (S4) of known alternaviruses. Further, this putative S4 appeared to be lost by serial subcultures, supporting that it is not strictly necessary for viral replication. Such loss of S4 during serial fungal passage without significant impact on the host morphology and growth was described before for other alternaviruses (Wen et al. 2021). Moreover, many alternaviruses lack an S4 completely (Table 3).

**Table 3:** Overview of the genome organization of all available alternaviruses to date with abbreviations, hosts, and GenBank accession numbers

Virus	Abbreviation	Refs	Original host	Segments	Segment length (bp)	Accession Nr	ORF (protein) size			Function	UTR (bp)	
							nt	aa	kDa		5′	3′
Dactylonectria torresensis alternavirus 1	DtAV1		Dactylonectria torresensis O6-1-A	3	dsRNA1 (3578)	OM296437	3375	1124	126.6	RdRp	62	141
					dsRNA2 (2668)	OM296438	2268	755	81.7	put. MTase	66	334
					dsRNA3 (2467)	OM296439	2214	737	79.7	CP	75	178
Ilyonectria robusta alternavirus 1	IrAV1		Ilyonectria robusta O16-2-D	3	dsRNA1 (3529)	ON721403	3375	1124	126.3	RdRp	80	74
					dsRNA2 (2464)	ON721404	2271	756	83.7	put. MTase	77	116
					dsRNA3 (2469)	ON721405	2232	743	81.6	CP	77	160
Fusarium graminearum alternavirus 1	FgAV1	He et al. 2018)	*Fusarium graminearum AH11*	3	dsRNA1 (3524)	NC_036596	3372	1123	126.6	RdRp	80	72
					dsRNA2 (2470)	NC_036601	2271	756	83.8	put. MTase	79	120
					dsRNA3 (2460)	MG697236	2232	743	81.3	CP	77	105
Fusarium poae alternavirus 1	FpAV1	Osaki et al. 2016)	*Fusarium poae MAFF 240,374*	3	dsRNA1 (3559)	NC_030883	3372	1123	126.5	RdRp	82	105
					dsRNA2 (2473)	NC_030880	2271	756	83.7	put. MTase	82	120
					dsRNA3 (2462)	NC_030881	2232	743	81.3	CP	77	59
Fusarium incarnatum alternavirus 1	FiAV1	Zhang et al. 2019)	*Fusarium incarnatum LY003-07*	3	dsRNA1 (3525)	MH899114	3372	1123	126.5	RdRp	82	71
					dsRNA2 (2469)	MH899115	2271	756	83.9	put. MTase	80	118
					dsRNA3 (2451)	MH899116	2220	739	80.7	CP	78	153
Aspergillus mycovirus 341	AMV	Hammond et al. 2008)	*Aspergillus niger* 341	4	dsRNA1 (3571)	EU289897	3375	1124	126.9	RdRp	51	145
Aspergillus foetidus mycovirus	AfMV	Kozlakidis et al. 2013)	*Aspergillus foetidus IMI 41,871*	4	dsRNA1 (3571)	NC_020103	3375	1124	126.9	RdRp	51	145
					dsRNA2 (2734)	NC_020100	2406	801	87.4	put. MTase	48	185
					dsRNA3 (2418)	NC_020101	2181	726	78.9	CP	50	187
					dsRNA4 (1961)	NC_020102	1743	580	65	unknown	50	168
Aspergillus heteromorphus alternavirus 1	AheAV1	Gilbert et al. 2019)	*Aspergillus heteromorphus* CBS 117.55	3	dsRNA1 (3559)	MK279437	3375	1124	126.9	RdRp	42	142
					dsRNA2 (2734)	MK279438	2502	833	91.4	put. MTase	48	184
					dsRNA3 (2417)	MK279439	2184	727	78.7	CP	49	184
Fusarium solani alternavirus 1	FsAV1	Lutz et al. 2022)	*Fusarium solani* NW-FVA 2572	4	dsRNA1 (3484)	OM326757	3387	1128	126.3	RdRp	53	44
					dsRNA2 (2595)	OM326758	2496	831	90.7	put. MTase	53	46
					dsRNA3 (2349)	OM326759	2187	728	78.5	CP	52	110
					dsRNA4 (1692)	OM326760	1224	407	43.9	unknown	186	282
Cordyceps chanhua alternavirus 1	CcAV1	Zhang et al. 2022)	*Cordyceps chanhua RCEF6000*	3	dsRNA1 (3488)	OK481552	3384	1127	126.4	RdRp	48	56
					dsRNA2 (2632)	OK481553	2496	831	90.7	put. MTase	51	85
					dsRNA3 (2369)	OK481554	2196	731	78.7	CP	53	41
Fusarium oxysporum alternavirus 1	FoAV1	Wen et al. 2021)	*Fusarium oxysporum BH19*	4	dsRNA1 (3424)	MT659125	2757	918	103.7	RdRp	628	39
					dsRNA2 (2677)	MT659126	2523	840	93.1	put. MTase	65	49
					dsRNA3 (2382)	MT659127	2190	729	78.7	CP	70	86
					dsRNA4 (1873)	MT659128	1167	388	42.9	unknown	151	496
Stemphylium lycopersici mycovirus	SlV	[DS]	Stemphylium lycopersici	4	dsRNA1 (3564)	NC_040515	3447	1148	128.9	RdRp	50	67
					dsRNA2 (2750)	NC_040516	2529	842	90.8	put. MTase	59	162
					dsRNA3 (2543)	NC_040518	2271	756	80.2	CP	60	212
					dsRNA4 (1398)	NC_040517	1194	397	41.1	unknown	35	169
Alternaria alternata dsRNA mycovirus	AaV-1	Aoki et al. 2009)	*Alternaria alternata EGS 35–193*	4	Segment L (3567)	NC_010984	3450	1149	129.3	RdRp	48	69
					Segment M1 (2744)	NC_010989	2535	844	90.7	put. MTase	52	157
					Segment M2 (2540)	NC_010990	2280	759	82.1	CP	51	209
					Segment S (1382)	NC_010991	1182	393	41	unknown	50	150

*DS* direct submission, *RdRp* RNA-dependent RNA-polymerase, *CP* Coat protein, *put. MTase* putative Methyltransferase

The segments discovered in the dsRNA of *I. robusta* have a total length of 3529 bp, 2464 bp, and 2469 bp, corresponding to the estimated size of the bands visible in the agarose gel (Fig. 1b). These segments were assigned to a putative new member of the proposed family *Alternaviridae*, which was tentatively named ilyonectria robusta alternavirus 1 (IrAV1), as the first member of the proposed new species *Alternavirus ilyonectriae*.

RT-PCR assays were developed to amplify the genomic fragments of the two new alternaviruses, with amplicons of expected fragment sizes of 846 bp, 548 bp, and 561 bp for DtAV1 and 943 bp, 622 bp, and 625 bp for IrAV1 (Fig. 1a). Sequencing of all amplicons confirmed the presence of DtAV1 and IrAV1, respectively, underlining the specificity of the primers (data not shown). Viral infection could be verified in the fungal isolates grown in culture over multiple generations.

### Genome organization

Both viruses DtAV1 and IrAV1 possess a genome composed of three dsRNA segments with a single ORF each and poly (A) tails at the 3′ end. DtAV1 has a total genome size of 8713 bp with a 3578 bp segment 1 (S1) encoding a 126.6 kDa RdRp on a 3375 bp open reading frame (ORF), flanked by a 62 bp 5′ UTR and a 141 bp 3′ UTR. A conserved RdRp_4 (pfam02123) site was found on aa position 641–754 with a confidence *E*-value of 0.002 calculated with NCBI-CDD search. Segment 2 (S2) has a length of 2668 bp with a 66 bp-long 5′ UTR and a 334 bp-long 3′ UTR. ORF 2 encodes an 81.7 kDa protein with a so far unknown function. The third fragment has a total length of 2467 bp with a 75 bp and a 178 bp UTR at the 5′- and 3′-end, respectively. The ORF of this segment encodes the 79.7 kDa major coat protein, which was identified, based on the study by (Wu et al. 2021).

**Fig. 2:**
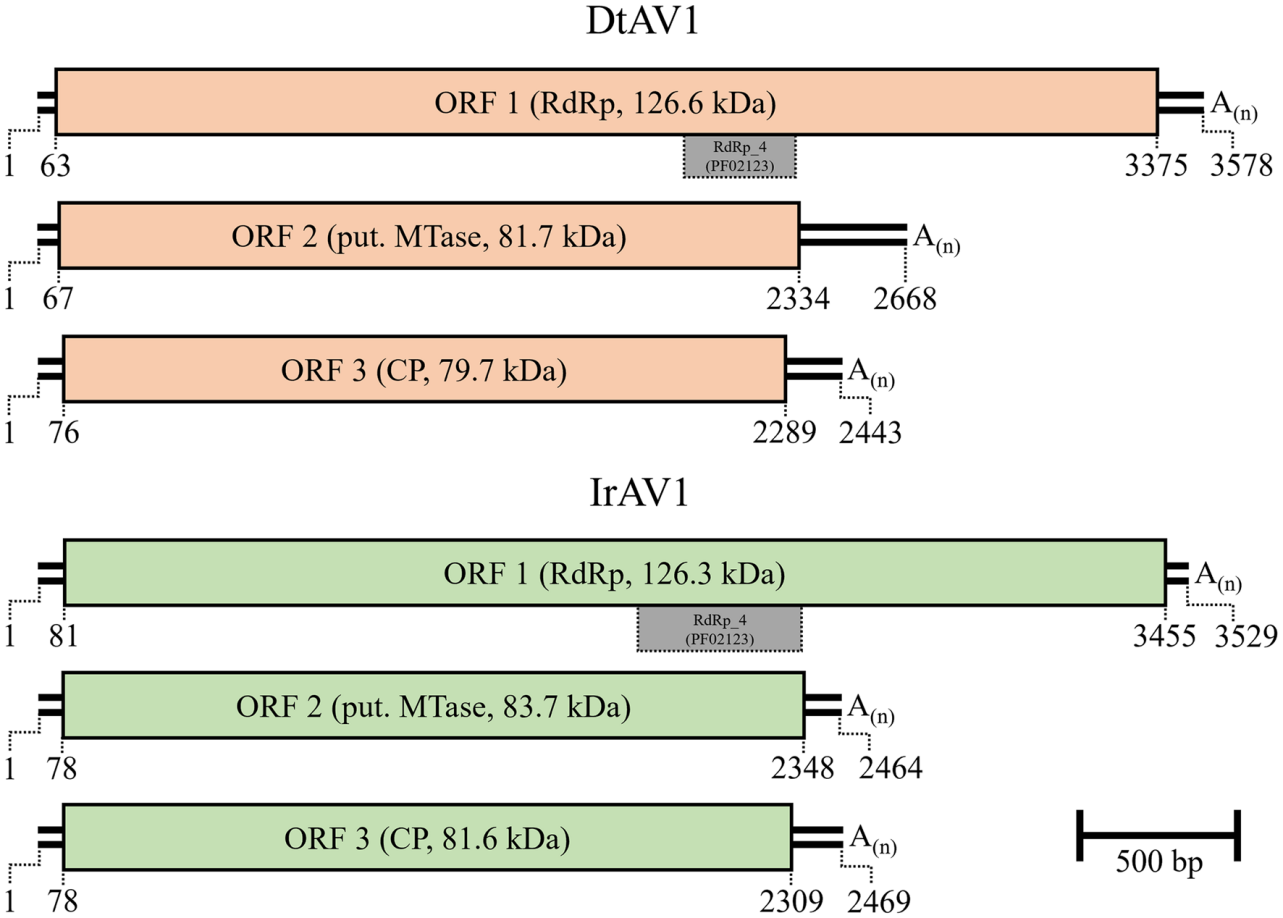
Scaled genome map of the identified viruses dactylonectria torresensis alternavirus 1 (DtAV1, light red) and ilyonectria robusta alternavirus 1 (IrAV1, light green). Double lines:genomic dsRNA. Digits: nucleotide positions of 5′-ends, ORFs and 3′-ends. Boxes: ORFs with the respective encoding protein and protein size in brackets. Grey boxes: Conserved RdRp domains. A_(n)_: Poly(A)-tail. Scale bar: 500 bp. “RdRP” = RNA-dependent RNA-polymerase; “put. MTase” = putative Methyltransferase; “CP” = Coat protein

IrAV1 has a total genome size of 8462 bp. S1 is 3529 bp long and encodes a 126.3 kDa RdRp on a 3375 bp ORF, flanked by an 80 bp 5′ UTR and a 74 bp 3′ UTR. A conserved RdRp_4 (pfam02123) site was found on aa position 587–753 with an E-value of 0.046. S2 has a total length of 2464 bp with a 77 bp-long 5′ UTR and a 116 bp-long 3′ UTR. The ORF encodes an 83.7 kDa protein with a so far unknown function. The third fragment has a total length of 2469 bp with a 77 bp and a 160 bp UTR at the 5′- and 3′-end, respectively. The ORF of this segment encodes the 81.6 kDa major coat protein. A scaled genome map of both viruses is shown in Fig. 2.

**Fig. 3:**
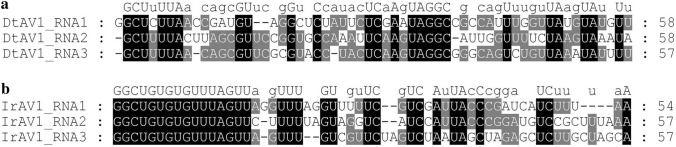
Alignments of 5 ‘ UTRs of **a** Dactylonectria torresensis alternavirus 1 (DtAV1) and **b** Ilyonectria robusta alternavirus 1 (IrAV1). Consensus sequences are shown above the alignment. Digits: Position of last shown nucleotide. Conservation indices: Black = 100%; grey ≥ 66.67%. Displayed with the GeneDoc software

The analyses of conserved 5′ end lead to diverse results when comparing DtAV1 and IrAV1. For IrAV1, the first 16 nucleotides (5′-GGCUCUCUCUUUAGUU-3′) are conserved in all three fragments. When aligning the 5′ ends of DtAV1 the segments are far more variable with 8 common nucleotides (5′-GCUYUUAM-3′) and an additional G as the first nucleotides of segment 1. The 5′ UTR alignments are shown in Fig. 3.

**Fig. 4:**
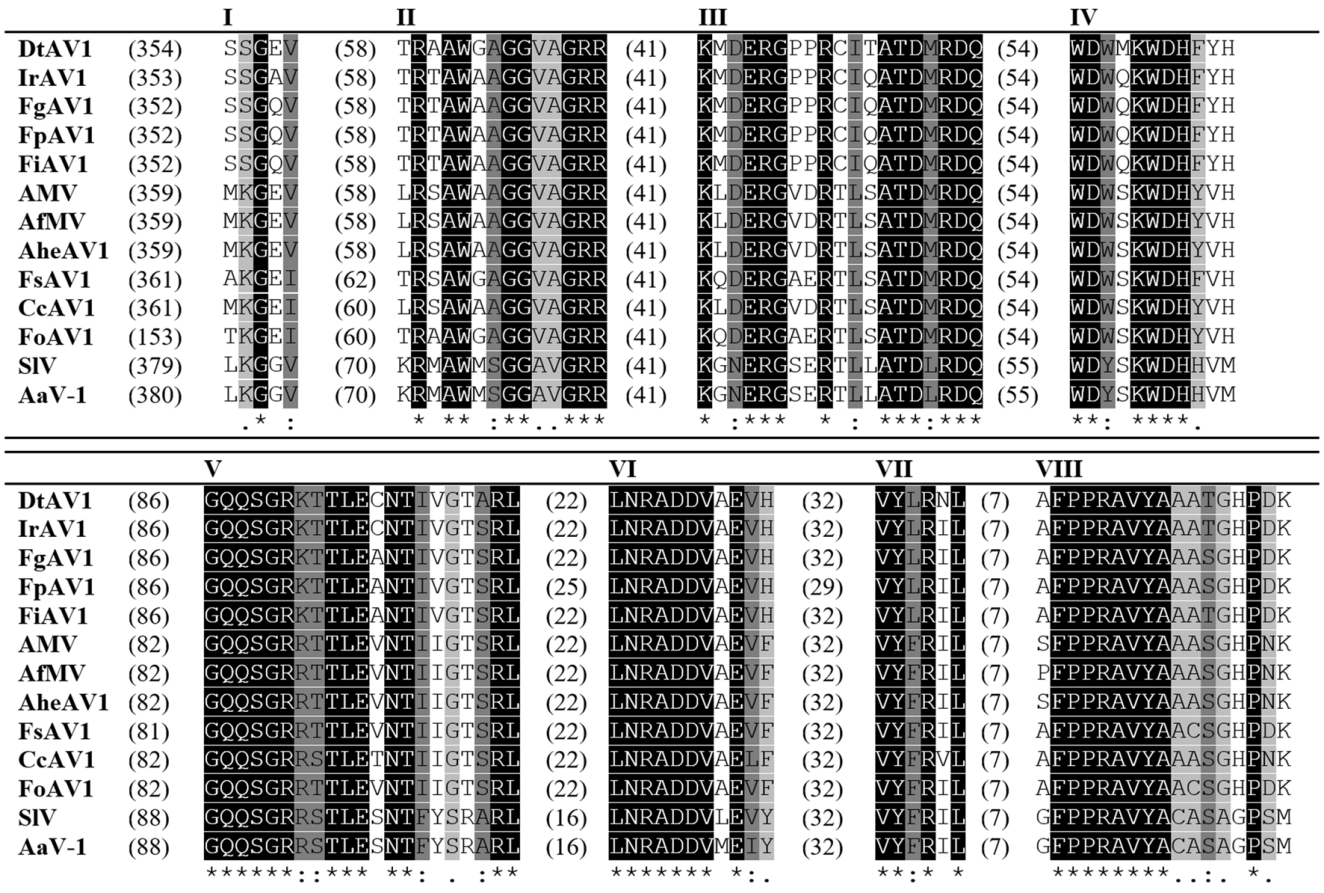
Alignment of conserved RdRp motifs I–VIII of available alternaviruses according to Gilbert et al. 2019, calculated with Clustal Omega (Gilbert et al. 2019; Sievers et al. 2011). Digits in brackets: Unlisted amino acids. Indices: Asterisk (black): Full conservation; colon (dark grey): Conservation of aa with strongly similar properties (> 0.5 [Gonnet PAM250]); period (light grey): Conservation of aa with weakly similar properties (> 0, =  < 0.5 [Gonnet PAM250]) (Gonnet et al. 1992)

Since the 5′ RACE of DtAV1 provided clear results, the 5′ terminal nucleotides of the other alternaviruses were aligned to compare the results. These alignments ranged from high conservation (e.g., CcAV1, FoAV1, IrAV1) to high diversity (e.g., FpAV1, SlV, AheAV1). Therefore, we conclude that conserved 5′ ends are no common feature of alternaviruses. Nevertheless, the conserved RdRp motifs I–VIII were identified by alignments according to Gilbert et al. 2019 for all available alternaviruses and the results are summarized in Fig. 4 (Gilbert et al. 2019).

The crucial metal ion binding ADD triplet in motif VI is rather unusual, as most of the RNA viruses share a GDD or SDD at this position (Venkataraman et al. 2018). DtAV1 and IrAV1 both match the conserved areas with the other alternaviruses in the alignment.

### Determination of putative protein functions

After the identification of the major coat protein on segment 3 (S3), the functions of two of the three segments are decrypted for DtAV1 and IrAV1 (Wu et al. 2021). With this information and BLASTp alignments, the CP was identified for all available alternaviruses on S3 regardless of whether the respective virus is tri- or quadripartite. A full overview of the current status of the genome organization of all alternaviruses is shown in Table 3.

To assign a putative function of the protein encoded by S2, further bioinformatic analyses were performed. Since Wu et al. 2021 described a methylated cap at the 5′ ends of the alternaviruses analyzed in that study, it is plausible that other alternaviruses possess capped RNAs as well (Wu et al. 2021). Terminal structures like m^7^G caps and polyadenylated 3′-ends were reported several times for dsRNA viruses (Aoki et al. 2009; Furuichi and Miura 1975; Wei and Moss 1975). Nevertheless, the simultaneous occurrence of both structures is very rare (Wu et al. 2021). Caps and polyadenylation of mRNAs usually serve to recruit RNA by translation factors and increase translation efficiency. In addition, the terminal structures protect against degradation by exonucleases (Gallie 1991). As for cellular mRNA, this also applies to viruses that take evolutionary advantage of these terminal structures (Wu et al. 2021; Furuichi and Miura 1975; Wei and Moss 1975; Schneider and Mohr 2003). So far, three mechanisms for capping viral RNAs are known. In the first case, mRNA capping is usually dependent on the host RNA polymerase II (Schneider and Mohr 2003). However, this is rather unlikely for the alternaviruses, since they are transcribed by their own RdRp, and RNA pol II is DNA dependent. The second scenario is cap-snatching, where viral RNAs take over the 5' caps of cellular mRNAs, by the activity of viral endonucleases (Wu et al. 2021; Schneider and Mohr 2003; Fujimura and Esteban 2011). As we were not able to identify conserved domains or motifs of known viral endonucleases in the genomes of DtAV1 or IrAV1, neither in the ORF of S2 nor in segments 1 and 3, we conclude that it is improbable that alternaviruses are able to reutilize mRNA caps from host transcripts. This fact suggests that it is likely that alternaviruses would use the third capping option and encode their own capping proteins. In the case of an m^7^G cap, as shown by Wu et al. 2021, a viral MTase would be required (Wu et al. 2021; Schneider and Mohr 2003).

**Fig. 5:**
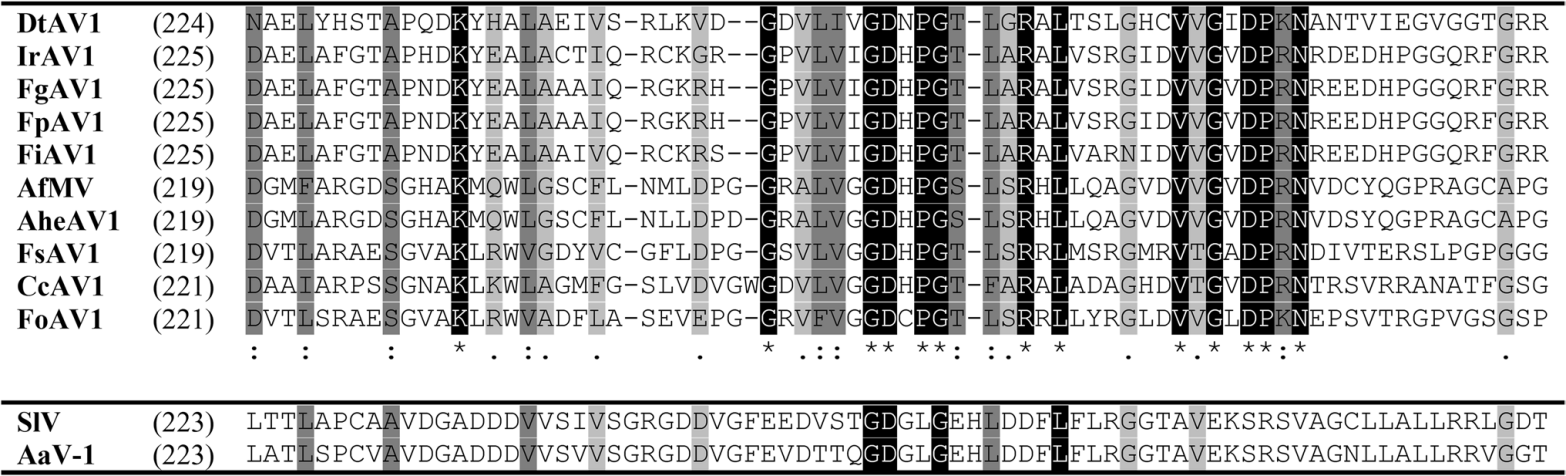
Alignment of conserved MTase (methyltransferase)-motifs on dsRNA2 of available alternaviruses, calculated with Clustal Omega (Sievers et al. 2011). Digits in brackets: unlisted amino acids. Indices: Asterisk (black): full conservation; colon (dark grey): conservation of aa with strongly similar properties (> 0.5 [Gonnet PAM250]); period (light grey): conservation of aa with weakly similar properties (> 0, =  < 0.5 [Gonnet PAM250]).”SlV” and “AaV-1” were not taken into account in the calculation of the indices

By evaluating the output of the function predictions for the protein with so far unknown function with ColabFold, followed by DeepFRI of DtAV1 and IrAV1, we received evidence of a possible RNA-binding domain for both viruses (Mirdita et al. 2022; Gligorijević et al. 2021). The highest-scoring molecular functions are heterocyclic compound binding (GO:1901363), organic cyclic compound binding (GO:0097159), nucleotide binding (GO:0000166), and nucleoside phosphate binding (GO:1901265). The template modeling scores (TM-scores), which evaluate how trustworthy the results are, range from 0.79 to 0.89 for IrAV1 and 0.94 for DtAV1. When evaluating the DeepFRI algorithm, Gligorijević et al. found TM-scores > 0.5 to be significant and scores > 0.73 to be very specific (Gligorijević et al. 2021). Since these results are giving reliable hints for a possible MTase activity of a protein encoded by S2, the amino acids sequences were screened for possible MTase domains by searching for templates with the SWISS-MODEL server (Waterhouse et al. 2018). The best matches of IrAV1 S2 were found within aa 233 to aa 286. Analyses of this region revealed a high identity (34.78%) with a heterocyclic toxin methyltransferase (SWISS-MODEL Template Library ID (SMTL ID): 7bgg.1.A). For DtAV1 S2, the best matches were found in aa position 233–285. Searching for templates within the section resulted in the best matches with the same MTase (7bgg.1.A) and an identity of 36.36%. These results suggest that (i) DtAV1 and IrAV1 may have a methyltransferase function in vivo and (ii) components of the necessary machinery are possibly encoded by S2. By considering these results, it was possible to identify a putative MTase domain in the ORF of every available S2 within the group of alternaviruses. To determine conserved motifs of the putative MTase domains, an alignment was computed (Fig. 5). Since the domains of SlV and AaV-1 stand out in comparison to the other viruses, they were considered separately and not included for the determination of conserved regions. The special position of these two viruses can also be observed in the phylogenetic results. However, possible methyltransferase domains were also found for these viruses. These showed the best matches with a cobalt-precorrin-6Y C(15)-methyltransferase (2yxd.1.A).

According to the sequence of DtAV1, the regions from aa 258 to aa 268 (GDXPG[T/S][L/F][G/A/S]RXL) and aa 275 to aa 282 (V[V/T]GXDP[K/R]N) stand out with high conservation. Performing a blastp search using aa 259 to aa 283 (GDHPGTLARALVSRGIDVVGVDPRN) of IrAV1 S2, containing the conserved sequences indicated above, delivered expected hits for ORF2 of alternaviruses and for more than 40 classI SAM-dependent metyhltransferases of bacteria, further strengthening the hypothesis for a methyltransferase function of the ORF of dsRNA2. However, since all of these results were generated via in silico calculations, it is crucial that future studies confirm the methyltransferase function with in vitro assays before it can be unequivocally assumed that alternaviruses indeed encode such a function on dsRNA2. Other possible methods to confirm the protein function would be docking with ligands or molecular dynamics studies (Kar et al. 2017). Thus, by providing a strong hypothesis, this study provides the basis for targeted protein function analyses in follow-up studies.

### Phylogenetics

To confirm DtAV1 and IrAV1 as members of the proposed family *Alternaviridae*, different phylogenetic analyses were performed in the course of this study. Both the RdRp and CP amino acid sequences of all available alternaviruses were analyzed and maximum likelihood trees, as well as pairwise sequence identity matrices, were calculated.

**Fig. 6:**
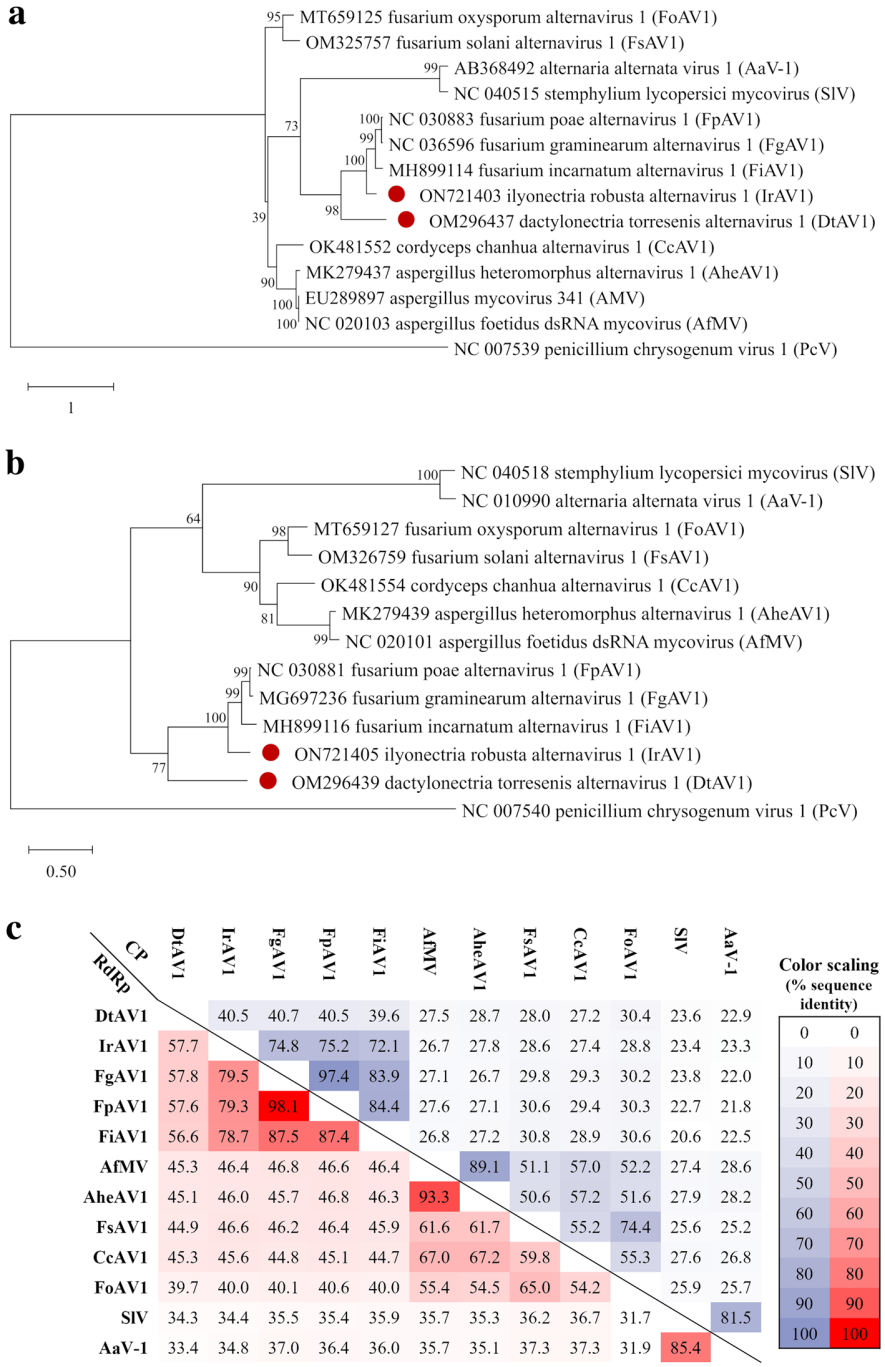
Phylogenetic trees and pairwise sequence identity matrix of available alternaviruses based on RdRp and CP amino acid sequences. Alignments were performed with the MUSCLE algorithm (Edgar 2004). Trees were constructed with the Maximum likelihood method and 1000 bootstraps in MEGA X (Kumar et al. 2018). All viruses are annotated with GenBank accession number. Colored dots indicate the viruses identified in this study. Scale bars representing substitutions per site. Numbers next to the branches are the percentage of trees, bootstrapped as shown. **a**: RdRp-based tree; substitution model: LG + G + F. **b**: CP-based tree; substitution model: WAG + G + F. **c**: Pairwise identity matrix of RdRp (red) and CP (blue) sequences, calculated with the EMBOSS/Needle tool with preset settings*.* Values are given in %. The scaling represents the color gradation of values from 0 to 100%

Within the RdRp-based tree (Fig. 6a), four major clusters are formed. DtAV1 and IrAV1 are clustering together with FpAV1, FgAV1, and FiAV1. All these viruses are originally hosted by Nectriaceae fungi. The second cluster is composed of AheAV1, AMV, AfMV, and CcV1, originating from *Aspergillus sp.* or *Cordyceps sp.* Species, respectively. Two smaller groups are built from AaV-1 with SlV and FoAV1 with FsAV1. These results confirm those from other studies and extend the phylogenetic knowledge of the proposed family *Alternaviridae* (Zhang et al. 2019, 2022).

In comparison to that, the CP-based tree is composed of 3 major clusters. As in the RdRp-based tree, DtAV1 and IrAV1 are clustering with FpAV1, FgAV1, and FiAV1. In contrast to the RdRp tree, FoAV1 and FsAV1 are summed up in a bigger cluster together with CcAV1, AheAV1, and AfMV (Fig. 6b). After the calculation of percentage pairwise sequence identities of the amino acid sequences of both the RdRp and CP a comparative double matrix was generated (Fig. 6c), to show that DtAV1 and IrAV1 have the highest sequence identity with FgAV1 in both protein sequences.

The clustering observed in the CP-based phylogenetic tree shows up similarly in the matrices. IrAV1 builds a cluster together with FpAV1, FgAV1, FiAV1, and DtAV1, which is slightly outside the group. FsAV1 and FoAV1 have the highest identity but contrary to the phylogenetic trees, they do not build a distinct group and are rather combined in one cluster with AfMV, AheAV1, and CcAV1. SlV and AaV-1 are building an outstanding group as observed before.

In summary, the RdRp-based tree appears to be sharper resolved, however, the clustering of the CP-based tree and the sequence identity matrix are more similar. Since most dsRNA mycoviruses, such as chrysoviruses and megabirnaviruses are distinguished by the phylogenetic analysis of RdRp and previous studies on alternaviruses have used the same approach, we recommend to continue to follow this criterion in the future (Aoki et al. 2009; Gilbert et al. 2019; Kozlakidis et al. 2013; Osaki et al. 2016; Wen et al. 2021; Zhang et al. 2019, 2022; Kotta-Loizou et al. 2020; Sato et al. 2019).

## Conclusions

In this study, two new mycoviruses were discovered and assigned to two new proposed species in the family *Alternaviridae*. We were further able to show that conserved 5′ ends do not exist for every virus and the RdRp motifs I–VIII are conserved with an active ADD triplet in motif VI for all alternaviruses. Further, as we showed that the ORF of S2 putatively encodes crucial motifs of a methyltransferase, it is possible that alternaviruses employ self-encoded proteins to cap their RNAs at the 5′ ends. Subsequent studies are crucial to confirm this hypothesis by specific work on segment S2 with techniques such as fusion-protein assays, protein–ligand docking, molecular dynamics or protein crystallization and X-ray analyses. The function of S3 could be inferred for all alternaviruses by BLAST alignments and the putative function of all confirmed ORFs were deciphered. After evaluation of the results of the phylogenetic analyses and the characteristics of the described viruses, we strongly support the assignment of the novel viruses to the proposed family *Alternaviridae*. The goal of this study was to get an insight into the virome of two ARD-associated endophytic fungi and to characterize the identified viruses in detail. To be of use for plant disease management, future studies need to focus on their biological influence by demonstrating whether there is a hyper- or hypovirulent effect of DtAV1 or IrAV1 on their hosts. These results will guide us to understand if the described alternaviruses could serve as possible biocontrol or mitigation agent to help counteract diseases like ARD.

## Supplementary Information

Below is the link to the electronic supplementary material.

Supplementary file1 (FASTA 4 KB)

Supplementary file2 (FASTA 3 KB)

Supplementary file3 (FASTA 2 KB)

Supplementary file4 (FASTA 3 KB)

Supplementary file5 (FASTA 2 KB)

Supplementary file6 (FASTA 2 KB)

Supplementary file7 (PDF 54 KB)

## Data Availability

Sequences will be available under the following NCBI GenBank accession numbers: Dactylonectria torresensis alternavirus 1 (DtAV1): OM296437–OM296439, Ilyonectria robusta alternavirus 1 (IrAV1): ON721403–ON721405
