# Supplementary material for: Molecular characterization of two new alternaviruses identified in members of the fungal family Nectriaceae
Source: Arch Microbiol. 2023 Mar 22;205(4):129. doi: 10.1007/s00203-023-03477-0 (PMC10033562; doi:10.1007/s00203-023-03477-0)
Supplement: Supplementary file 7 — Supplementary file7 (PDF 54 KB) [file 203_2023_3477_MOESM7_ESM.pdf]

Article title:

Molecular characterization of two new alternaviruses identified in members of the fungal family Nectriaceae

Journal:

Archives of Microbiology

Authors:

Tom P. Pielhop, Carolin Popp, Sebastian Fricke, Dennis Knierim, Paolo Margaria, Edgar Maiß

Corresponding author:

Tom P. Pielhop, pielhop@ipp.uni-hannover.de

Institute of Horticultural Production Systems, Dept. Phytomedicine, Leibniz University Hannover, Herrenhäuser Str. 2, 30419, Hannover, Germany.

Tab. ESM1: Number of reads mapped to the assembled contigs

Sample	Virus	Contig	Length (nt)	Mapped reads	% of mapped reads
O6-1-A	DtAV1	DtAV1_dsRNA1	3576	725	0.177
		DtAV1_dsRNA2	2648	1489	0.364
		DtAV1_dsRNA3	2451	3410	0.834
O16-2-D	IrAV1	IrAV1_dsRNA1-5'-region	1762	137	0.029
		IrAV1_dsRNA1-3'-region	1048	113	0.024
		IrAV1_dsRNA2	2428	324	0.068
		IrAV1_dsRNA3-5'-region	978	117	0.025
		IrAV1_dsRNA3-3'-region	1360	116	0.025
